# Application of Modified Natural Zeolite—Clinoptilolite for Bacterial Control in the Environment

**DOI:** 10.3390/ma18102411

**Published:** 2025-05-21

**Authors:** Jasna Hrenović, Nevenka Rajić

**Affiliations:** 1 Faculty of Science, Division of Microbiology, University of Zagreb, 1000 Zagreb, Croatia; jasna.hrenovic@biol.pmf.hr; 2 Faculty of Ecology and Environmental Protection, University Union Nikola Tesla, 11000 Belgrade, Serbia

**Keywords:** bacteria, zeolitized tuff, antibacterial activity, bactericidal activity

## Abstract

Natural zeolites are highly effective adsorbents that can remove various metal cations which would otherwise contaminate the environment. However, different metal cations (Cu, Zn, and Ag) within their lattice or quaternary long-chain surfactant cations on their surface modify their affinity towards hazardous anions and promote antibacterial activity in natural zeolites. Specifically, natural zeolites in their non-modified form lack intrinsic antibacterial characteristics. NZ is the most widespread natural zeolite. This review presents the antibacterial efficiency of NZ containing transition metals, nano oxides, and organics. This effect is nonspecific and primarily driven by the nutritional makeup of the medium rather than the species of pathogenic bacteria under study. Studies on using NZ-based disinfectants to clean up contaminated water and soil and using modified and purified NZ to protect health are also considered. By eliminating toxic ions and, when modified by these toxic cations, removing pathogens from the environment, natural NZ can serve a dual function, providing it with the distinctive characteristics of a sustainable material.

## 1. Introduction

In the 21st century, humanity confronts pollution across all components of the natural environment. Bacteria that are resistant to antibiotics and disinfectants are present in the environment, where they pose a public health risk, as a result of the dissemination of pathogenic bacteria through human solid and liquid waste [1,2].

Alternative agents that would leave a less harmful residue footprint are needed to control human pathogens in the environment.

Natural zeolitized tuffs themselves have no antibacterial activity. Moreover, zeolite particles are very suitable abiotic surfaces for attaching various bacteria that form stabile bacterial biofilm around it [3]. On the other hand, synthetic zeolites of Lynde type A act as antibacterial agents and are even genotoxic to eukaryotic yeast cells [4]. However, the wide use of synthetic zeolites A for disinfection is not common due to the hydrolysis of particles in water with a half-life of 1 to 2 months [5] and consequent leaching of aluminosilicate molecules that are toxic to all organisms. The antibacterial effects of commercial synthetic zeolite structures, FAU, LTA, and MFI, infused with silver, copper, or zinc ions were reported [6]. Silver-containing zeolites showed antimicrobial activity against *Escherichia coli*, methicillin-sensitive *Staphylococcus aureus*, and methicillin-resistant clinical isolate of *S. aureus*, suggesting the potential application of metal ion-exchanged zeolites in managing bacterial infections, particularly those caused by emerging antibiotic-resistant strains.

When zeolite is treated with established antibacterial agents, the predominantly non-toxic zeolite particles develop antibacterial characteristics. A plethora of scientific papers address the antibacterial activity of functionalized zeolites. Their primary limitation is the lack of a universally recognized definition of “antibacterial activity”. In scientific contexts, antibacterial activity denotes the mass concentration of functionalized zeolites that, following statistical analysis, significantly (*p* ≤ 0.05) diminishes the survival or proliferation of bacteria compared to the positive control (absence of material) [7].

Various terms are employed to describe antibacterial activity. Bactericidal activity refers to the concentration of a substance that effectively eliminates all bacteria present in the experimental conditions. Determining the minimum concentration of a substance that exhibits antibacterial or bactericidal properties is often beneficial. The objectives encompass minimum inhibitory concentration (MIC) and minimum bactericidal concentration (MBC). MIC represents the minimal concentration of a substance that inhibits the observable proliferation of bacteria following incubation in liquid media. The presence of zeolite particles in fluid media contributes to turbidity, thereby raising questions regarding the determination of MIC. MBC represents the minimal concentration of a substance required to eradicate all bacteria present in the medium [8]. Effective concentrations, including EC50 and EC90, are commonly utilized in ecological research. The specified concentrations exert a measurable effect on 50% or 90% of the examined population. The lethal dose LD50 quantifies the concentration required to cause mortality in 50% of a population [9].

In all the mentioned definitions, the antibacterial activity will depend on the initial number of bacteria. In clinical microbiology, this initial number is an inoculum of 5 × 10^5^ CFU (colony-forming units)/mL [10]. The antibacterial activity could also be standardized when using the standardized inoculum. Assuming that only a part of the whole volume subjected to the experiment would be analyzed, MBC indicates the concentration that kills 99.9% of the initial inoculum or lowers the bacterial number for ≥3 log_10_ CFU/mL.

Despite the lack of definition of antibacterial activity in all scientific fields, this review will assume the potential of functionalized natural zeolite—clinoptilolite to control bacterial concentration in the environment.

## 2. Structural Features of Natural Zeolites

Zeolites are naturally occurring or synthetic aluminosilicate solids, renowned for their unique three-dimensional framework structure. This framework contains well-defined pores and channels that enable the selective adsorption of molecules based on size and polarity. The pore sizes typically range from 0.3 to 1 nm, varying according to the specific type of zeolite. Typical natural zeolites include clinoptilolite, mordenite, and chabazite, each showcasing structural arrangements, pore sizes, and chemical compositions defining their specific applications.

**Figure 1:**
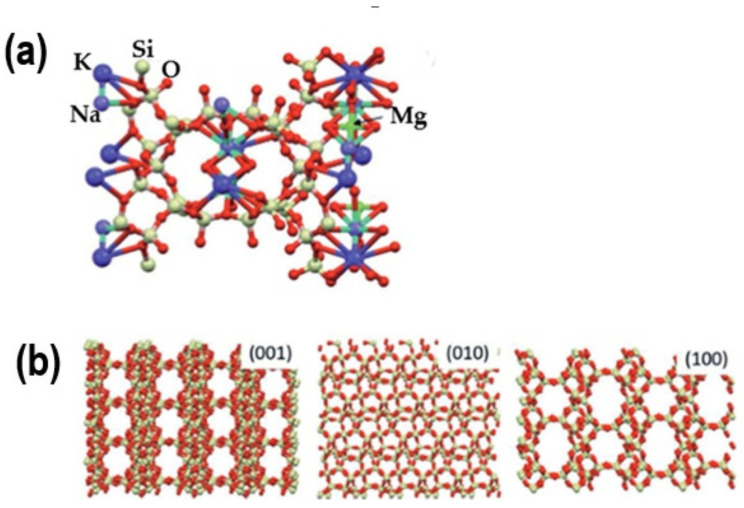
(**a**) Presentation of the clinoptilolite unit cell. Potassium, sodium, and magnesium are exchangeable cations. (**b**) Lattice structure along different crystallographic planes [11]; K—large blue balls; Na—petite blue balls; Mg—green balls; Si(Al)—pale green balls; O—red balls.

Natural clinoptilolite is distinguished by its crystalline structure, cation absorption, and exchange capacity. It possesses a three-dimensional framework composed of tetrahedra formed by silicon (Si) and aluminum (Al) atoms. Each tetrahedron comprises a core silicon or aluminum atom surrounded by four oxygen (O) atoms. These tetrahedra configurations form channels and cavities throughout the structure (Figure 1) [11].

Incorporating aluminum inside the framework generates a negative charge, which is neutralized by cations (such as Na^+^, K^+^, Ca^2+^, and Mg^2+^) situated in the channels and pores of the zeolite structure. This characteristic enables clinoptilolite to exchange cations, making it beneficial for water treatment and soil enhancement. Clinoptilolite possesses relatively wide pores and channels, typically measuring 0.3–0.4 nm in diameter, facilitating the selective adsorption of molecules according to their size and shape. This feature renders it advantageous for gas separation, ion exchange, and filtration applications [12,13,14,15,16].

Clinoptilolite exhibits thermal stability up to 800 °C (contingent on origin), enabling it to preserve its structural integrity during thermal processing such as drying or heating. The existence of hydroxyl groups and the inherently polar characteristics of the clinoptilolite structure render it hydrophilic, facilitating interactions with water molecules and other polar compounds [17,18,19,20].

Various cations can be selectively adsorbed according to their size, charge, and hydration energy, enabling clinoptilolite to be utilized in targeted applications such as selective ion exchange in water treatment. The structural characteristics of natural clinoptilolite facilitate its multifaceted applications in environmental cleanup, agriculture, construction, and several industrial operations [21].

## 3. Modification of Natural Clinoptilolite

Due to their microporosity, natural zeolites are suitable inorganic reservoirs for holding species with antibacterial activity and controlling their release [1,22,23]. Modifying clinoptilolite (NZ) with transition metal cations (such as Ag^+^, Cu^2+^, Zn^2+^), oxide nanoparticles (ZnO, TiO_2_, CuO or Cu_2_O), and organics (biopolymers, quaternary ammonium salts) enhances its intrinsic properties, facilitating applications in environmental science, catalysis, and materials engineering [24,25,26].

### 3.1. Modification with Transition Metal Cations

Transition metal ions (Cu^2+^, Zn^2+^, Fe^2+^/Fe^3+^, Co^2+^, Ni^2+^, and Ag^+^ with filled d subshell) exhibit antibacterial effects when applied in higher quantities. Their antibacterial activities contrast with those of antibiotics as they influence a broad spectrum of biomolecules, hence reducing the likelihood of bacterial resistance development. Specific metal ions may demonstrate heightened toxicity to target bacteria relative to humans and animals when employed as antibacterial agents. This mitigates the risk to human health, as the release of metal ions within the body may be controlled [27]. Inorganic antibacterial substances present several advantages compared to traditional organic agents. These include enhanced chemical stability, facilitating sustained antibacterial activity over extended periods, thereby offering prolonged protection against bacterial colonization. Additionally, they exhibit resistance to temperature and light, lower toxicity, a broader spectrum of activity, reduced likelihood of resistance development, and cost-effectiveness [28].

Replacing exchangeable cations (such as sodium or calcium) in NZ with antibacterial metal ions (e.g., silver, copper, zinc) through ion-exchange procedures imparts antibacterial properties to NZ [29]. The main advantage of using zeolites as a metal ion source is that the rigid zeolite structure preserves ions in their actual ionic state. This is particularly important for Ag(I), the primary active antibacterial agent. Furthermore, the microporosity of the zeolite framework enables regulated diffusion and controlled release of cations into external environments. A recent study demonstrated that the silver content in zeolite can be adjusted by modifying it with HCl or NaCl, which alters its adsorptive and ion-exchange properties. This modification allows for the regulation of silver content and distribution through changes in the ion-exchange capacity of the parent zeolite [30].

Na-enriched NZ from the Gordes deposit (Turkey) demonstrated selectivity for silver ions within the specified concentration range. Conversely, it exhibited a lack of selectivity for zinc and copper ions, except in low-concentration regions, where a slight selectivity of NZ was noted [31]. The antibacterial activities of the exchanged samples were assessed concerning the exchange level against *Pseudomonas aeruginosa* and *E. coli*, revealing that an increase in Ag loading did not enhance antibacterial activity. Consequently, Ag-containing NZ was proposed as a cost-effective antibacterial agent, given its selectivity sequence and antibacterial effectiveness [31].

The antibacterial activity of 1.0 wt% of Ni-, Cu- or Zn-exchanged NZ against *E. coli* DSM498 and *S. aureus* DSM799 was significantly lower in nutrient-rich Luria Bertani medium as compared to nutrient-poor simulative wastewater, where Ni-exchanged NZ had the worst efficiency [32]. In nutrient-rich peptone water, the Ag-exchanged NZ at 0.1 wt% removed 10^6^ CFU/mL of both *E. coli* DSM498 and environmental isolates of *E. coli* after one hour of contact, but Cu- and Zn-exchanged NZ were not efficient [33].

The Cu- and Ag-exchanged NZ were tested against 10^5^ CFU/mL of multidrug-resistant environmental isolates of *Acinetobacter baumannii* in diluted nutrient broth at room temperature. In all tests, MBC decreased with exposure time, and after 24 h, averaged 32–64 mg/L for Cu-exchanged or 0.25–2 mg/L for Ag-exchanged NZ [20]. The Cu- and Ag-exchanged NZ were also tested against 10^7^ CFU/mL of clinical isolates of *A. baumannii* in a physiological solution [34]. Again, MBC decreased with exposure time, and after 24 h at 36 °C averaged 125–250 mg/L for Cu-exchanged or 31–250 mg/L for Ag-exchanged NZ [35].

In the disinfection process, *E. coli* ATCC8739 and total coliform bacteria were used to determine microbiological contamination in water after using two Ag-exchanged NZ samples of different origins. Disinfection kinetics takes place in one or two steps, depending on the characteristics of the wastewater. The bactericidal effect on *E. coli* depends on the origin of the zeolite. The concentration of silver in the water after treatment ranged from 0.01 to 0.50 mg/L, depending on the NZ and the duration of disinfection. The silver amount in wastewater after treatment remains constant during the critical disinfection period [36].

In recent years, the inclination to diminish the presence of volatile organic components, including phenolic compounds and chloroacetamide, in contemporary buildings has produced and utilized a substantial array of water-based paints. Nonetheless, the aquatic environment conducive to microbial proliferation leads to the biodeterioration of coatings, plasters, and fundamental construction elements. To mitigate adverse effects, zeolites infused with transition metal ions (Ni(II), Cu(II), and Zn(II)) were investigated to produce gypsum composites for biocidal plasters and mortars. Antibacterial activity assessments against Gram-negative *E. coli* ATCC8739 and Gram-positive *S. aureus* ATCC6538 demonstrated that formulations containing Cu- and Zn-exchanged NZ exhibited a bacteriostatic effect against *E. coli*. At the same time, only Cu-zeolite was effective against *S. aureus* [36].

The removal of *E. coli* and heavy metals (Pb(II), Cd(II), and Zn(II)) from aqueous solutions using Ag-exchanged NZ was documented, involving the dual processes of *E. coli* disinfection by Ag^+^ ions and toxic metal adsorption on NZ. The Ag-containing NZ exhibited remarkable disinfection effectiveness, eliminating *E. coli* in 30 min. A removal effectiveness of 92% was achieved for the metals in the solution. In *E. coli*–metal ion solution systems, disinfection was enhanced by metal ions, resulting in a somewhat reduced the amount of metals adsorbed by the zeolites, as the bacterial cells in the solution absorbed the metal ions. The results of this study demonstrate that Ag-modified NZ can concurrently eliminate metals and pathogenic organisms from polluted water sources [37].

Bacteria-induced corrosion in sewage concrete pipes, collection systems, and treatment facilities is a global issue. Acid-resistant, high-performance coating materials like fiberglass, mortar, brick, ceramic, and polyurethane have been adopted to reduce costs. These materials impede corrosive substances in concrete, but they do not stop the proliferation of corrosive microorganisms, such as *Acidithiobacillus thiooxidans* and *Desulfovibrio desulfricans* [38]. The coating with Ag-exchanged zeolite was shown to be a low-cost alternative. This material inhibits the growth of planktonic and biofilm populations of *A. thiooxidans* [38].

The multidrug-resistant *A. baumannii* induces severe nosocomial infections. One method to control the infection is by managing biofilm formation in endotracheal tubes. To prevent this, PVC composites were synthesized by incorporating varying quantities of Cu(II)-exchanged NZ and impregnated with D-tyrosine (D-Tyr). The composites demonstrate an antibacterial effect on A. baumannii, attributed to the synergistic interaction between Cu-NZ and D-Tyr [39]. A silver- and zinc-containing zeolite matrix applied to stainless steel significantly enhances its antimicrobial properties. After 24 h, the materials reduced CFU by 100% and proved useful in settings where microbial contamination is undesirable [40]. Ag- and Zn-containing NZ developed long-term antimicrobial characteristics in paints and polypropylene, which increased with higher zeolite concentrations. It can be concluded that materials manufactured with metal-ion-exchanged zeolites could prevent microbial growth on surfaces, thereby reducing cross-contamination, infection risk, and product degradation [41,42].

The antibacterial effectiveness of transition metal-modified zeolites can be mainly attributed to the leaching of metal ions into the adjacent medium in contact with the zeolite [43,44,45]. The extent of leaching depends on the specific metal cations and the particular zeolite and media employed. Thus, in seawater, Ag–NZ and Cu–NZ demonstrated bactericidal effectiveness against *E. coli* and *A. baumannii* after 3 and 6 h of exposure, respectively, whereas the Zn–NZ showed antibacterial activity against both strains but did not exhibit a bactericidal effect even after 24 h of treatment. All studied samples discharged metal cations into seawater. The quantities of Ag and Zn leached from Ag–NZ and Zn–NZ were above the maximum allowable concentrations (MAC) for drinking water. However, the concentration of Cu was below MAC, indicating that Cu-NZ is an efficient disinfectant for seawater treatment. The high concentration of Na^+^ in salt water (about 1200 mg/L) largely enabled leaching via an ion exchange mechanism. The limited degree of leaching from Cu–NZ is likely attributable to Cu(II) occupying distinct locations within the NZ lattice and its geometric arrangement, which inhibits its release [46]. Moreover, Ag-exchanged zeolite integrated into a polypropylene polymer matrix exhibits considerable biocidal activity against *S. aureus*. Darkening due to diminished silver species limits its application, but it can be prevented by incorporating a photo stabilizer. Its addition did not compromise biocidal effectiveness and maintained the original color of the polypropylene polymer. The released Ag^+^ ions serve as the biocidal active agents, and they are liberated even when the Ag-zeolite is incorporated into the polymer, bestowing bactericidal capabilities to the resultant Ag-zeolite-polymer composite [47].

Several processes contribute to the antibacterial activity of metal ions, including (1) bacterial cell membrane rupture, which compromises cell integrity and substance transport. Metal ions can displace essential ions from bacterial cell membranes, and this displacement can disrupt cellular function, leading to bacterial death; (2) metal ion interaction with amino (-NH2), carboxyl (-COOH), and thiol (-SH) groups of amino acid residues, causing protein damage; (3) damage to DNA and nucleic acids; (4) the production of reactive oxygen species (ROS); or (5) the disturbance of metal ion homeostasis. High metal concentrations can break the delicate balance and induce toxicity by competing for sites, as bacteria need metal ions for essential processes [27].

**Figure 2:**
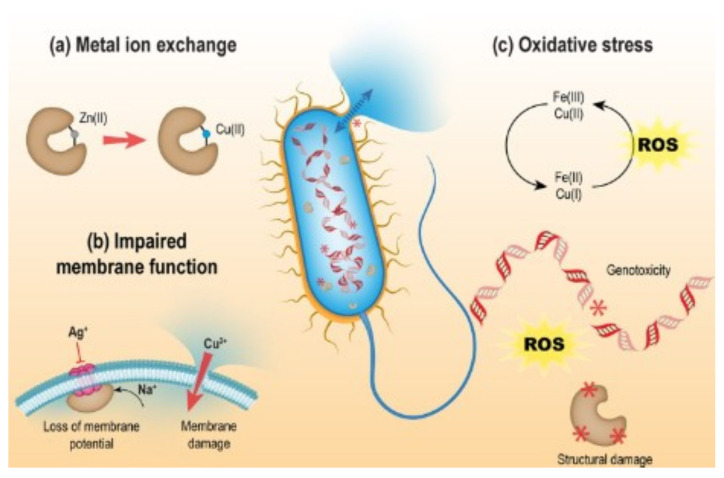
Possible mechanisms of antibacterial activity for different transition metal ions: (**a**) replacement of metal ions, (**b**) damage to bacterial membranes, and (**c**) oxidative stress; production of reactive oxygen species leading to the denaturation of genetic elements and proteins (Reproduced from ref. [27] with permission from the Royal Society of Chemistry).

Figure 2 illustrates the antibacterial activity of Zn(II) and Cu(II) ions. Metal ions compromise the cell membrane integrity and denaturation of biomolecules, including metalloproteins and DNA, leading to cell death. A mechanism for biomolecule denaturation involves oxidative damage caused by ROS, including hydroxyl radicals, superoxide anions, and singlet oxygen. These ephemeral species can be generated from O_2_ or H_2_O within the cell following the aggregation of metal ions implicated in single-electron transfer, such as Cu(II) ions. Although biological reactions lead to the neutralization of ROS and damage repair, excessive oxidation will cause cell death [27].

### 3.2. Modification with Nano Oxide Particles

Modifying NZ with metal oxide nanoparticles seems promising due to the lower leaching of metal cations than in NZ modified with metal ions. The leaching of metal cations from nano ZnO-supported NZ (0.25 wt%) and NiO-NZ (0.51 wt.%) is much lower than the leaching of Zn(II) (1.07–1.61 wt%) and Ni(II) ions (3.44–9.13 wt%) from Zn(II)- and Ni(II)-exchanged NZ, respectively [32]. This suggests higher stability of nanoparticles supported onto NZ than the metal ions present inside the zeolite lattice. Moreover, using metal oxide nanoparticles supported on zeolites has been a prominent strategy to overcome pathogenic microorganisms [48].

**Figure 3:**
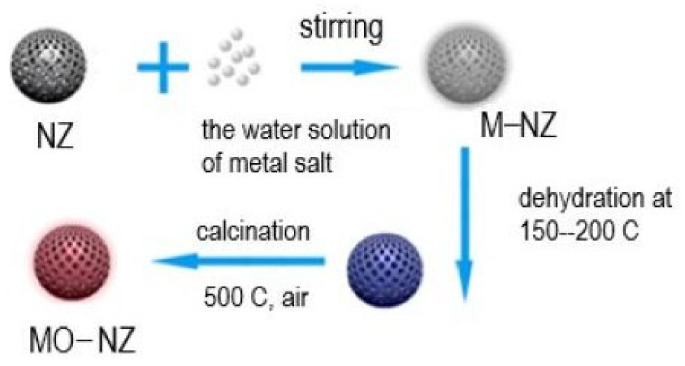
Schematic presentation of integrating nano oxides into the NZ framework (adopted from [52]).

Integrating nano oxides into the NZ framework (Figure 3) imparts antibacterial properties that are highly relevant in wastewater treatment, biomedical engineering, and materials research. Nano oxides (such as TiO_2_, ZnO, or CuO) possess a substantial surface area and numerous reactive sites that can engage with bacterial cells, enhancing antibacterial efficacy. Moreover, certain metal oxides gradually release metal ions (e.g., Ag^+^, Zn^2+^, Cu^2+^) with antibacterial characteristics. These ions can compromise bacterial cell membranes, obstruct metabolic processes, and result in cell death. When exposed to ultraviolet light, specific nano oxides, particularly TiO_2_, can produce ROS, which damage bacterial DNA, proteins, and lipids, leading to cellular demise. Generally, two mechanisms are proposed for the antimicrobial efficacy of nanoparticles: (a) toxicity from free metal ions resulting from the dissolution of metals from the nanoparticle surface and (b) oxidative stress through the production of ROS via hydrogen peroxide and organic hydroperoxides on the nanoparticle surface. Nanoparticles can influence the viability of microorganisms by agglomerating on bacterial surfaces and modifying the structure of lipids, peptidoglycan, proteins, and DNA [49]. The embedding of nanoparticles in the zeolite lattice controls their release and enhances their antimicrobial properties. Recently, the antimicrobial test of the zeolite-supported nanoparticles ZnO and TiO_2_ confirmed considerable antimicrobial activity against Gram-positive and harmful bacteria. Antimicrobial activity depends on the type of nanoparticles and the species of microorganism (Gram-positive or Gram-negative) [50]. The authors proposed that ZnO and TiO_2_ nanoparticles alter the cell wall’s and cell membrane’s permeability, consequently impacting biomolecules like DNA and proteins, and inhibiting processes such as DNA replication and protein synthesis [50].

The nano ZnO supported on zeolite demonstrated high antibacterial activity against the Gram-positive and Gram-negative bacteria, and the activity increased with an increase in the amount of ZnO NPs (3–8 wt.% ZnO). The bactericidal effect decreases according to the following sequence: *E. coli* E266 > *Bacillus subtilis* B29 > *Salmonella choleraesuis* ATCC 10708 > *S. aureus* S276. The enhanced antibacterial activity was attributed to surface defects on the ZnO, an abrasive surface texture, and the presence of zeolite support, which prevents the agglomeration of the nanoparticles [51]. The ZnO supported on a zeolite surface demonstrated superior antibacterial efficacy compared to nano-ZnO particles under visible light, particularly against *E. coli*. 

The average concentration of total ROS generated under visible light conditions from immobilized ZnO particles in aqueous suspension was much higher than that of nano-ZnO. The greater specific surface area of immobilized ZnO compared to nano-ZnO was suggested to be responsible for providing more accessible reaction sites. This led to the conclusion that supporting ZnO onto a zeolite surface is an excellent way to enhance the antibacterial properties of ZnO under visible light circumstances [52].

The NZ, after its utilization in the extraction of Cu(II), Zn(II), and Ni(II) ions from wastewater, was then employed in the disinfection of secondary effluent and the elimination of pathogenic bacteria during the tertiary phase of wastewater treatment. A straightforward calcination process transformed NZ into a carrier of nano oxides (NPs) (Figure 3). The antibacterial efficacy of NiO, Cu_2_O, and ZnO NPs supported on NZ depends on the specific oxides and the bacterial species involved. Cu_2_O- and ZnO-NZ exhibited exceptional antibacterial activity, whilst Cu_2_O and NiO-NZ demonstrated antiprotozoal activity after one hour of contact in the secondary effluent water. The nanoparticles remained stable during interaction with bacteria [53]. This shows a sustainable solution for wastewater treatment using NZ.

The leaching of Cu(II) ions from the zeolite lattice was impeded by converting Cu(II) ions into copper oxides (CuO and Cu_2_O). The antibacterial activity assay against *E. coli* revealed that the copper oxide/zeolite composite showed considerable antibacterial efficiency and exhibited more potential as a water filter than copper/zeolite. The Cu(II) concentration released from the synthesized materials was under 2 mg/L, indicating its safety for usage with a maximum contact length of less than 20 min or between 50 to 60 min [54]. Additionally, cupric oxide and cuprous oxide NPs are attracting interest for their antibacterial properties against Gram-positive and Gram-negative bacteria and their multitoxicity towards multidrug-resistant pathogens. They are less expensive and more plentiful than noble metals such as gold and silver [55]. Their stability is maintained through immobilization within the zeolitic lattice, preventing the aggregation of bigger clusters that result in a loss of reactivity [54,56].

NZ saturated with Cu(II) ions and coated with CuO nanoparticles, substantially inhibited the multiplication of methicillin-resistant clinical isolate of *S. aureus* and *Enterococcus faecalis* ATCC29212. The authors suggested that the ease of processing and cost-effectiveness of Cu(II)-zeolitic material might be employed for dental biomedical applications, either directly or as a bactericidal additive in 3D printing filaments [56]. The antibacterial efficacy of CuO-supported zeolite against *E. coli* and *S. aureus* is associated with both Cu^2+^ content and CuO particle size [57]. The antibacterial activity is attributed to the release of Cu(II) ions from nano oxide particles. The effect diminishes as nanoparticles agglomerate, inhibiting the leaching of Cu(II) ions [58,59]. The NZ infused with silver nanoparticles was tested in the suspension of *E. coli* ATCC25922 in Mueller–Hinton broth at a concentration of 10^4^ CFU/mL. After 24 h of contact at 35 °C, MIC and MBC of 1.56 g/L were obtained [60]. The concentration of silver nanoparticles immobilized onto NZ required to exhibit bactericidal activity against *E. coli* was fourfold lower than that of silver nanoparticles alone. The NZ functionalized with gold nanoparticles removed up to 95% of *E. coli* and *Salmonella typhi* (bacterial strains were not defined) from Tripticaseine broth after 90 min of contact at 35 °C [61]. The antibacterial activity of NZ modified with metal ions or metal oxide nanoparticles is influenced by several factors, including the type of metal cations used for modification, the concentration of functionalized NZ, the initial concentration of target bacteria, the duration of contact with bacteria, the nutritional composition, and the temperature of the water medium [60,61].

### 3.3. Modification with Organic Species

Coating NZ with biocompatible polymers like chitosan or polyvinyl alcohol, known for their inherent antibacterial properties, enhances the antibacterial efficacy of NZ. Infusing natural antibacterial agents, such as essential oils (e.g., tea tree oil, eucalyptus oil) or botanical extracts (e.g., garlic extract, oregano oil), into the NZ lattice brings the antibacterial properties to NZ. Incorporating synthetic substances, such as quaternary ammonium compounds, can yield substantial antibacterial properties. The incorporation of amine or carboxyl groups on the surface of NZ enhances its interaction with bacteria. Amino-functionalized zeolites exhibit notable antibacterial activity, whereas carboxylation enhances bacterial contact, thereby augmenting antibacterial efficacy. The formulation of NZ composites with antibacterial polymers yields materials that demonstrate improved antibacterial properties, making them suitable for diverse applications, including medical use [58].

**Figure 4:**
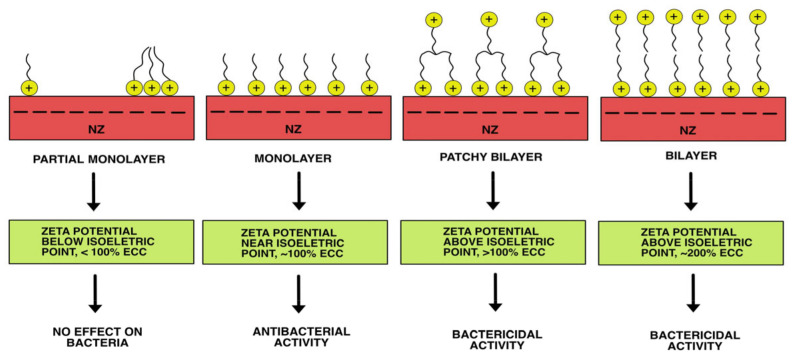
Antibacterial activity depends on the configuration of cationic surfactant molecules adsorbed onto NZ.

Cationic surfactants are well-known antibacterial agents widely used for disinfection and antisepsis, with an EC50 of around 1 mg/L [62]. Cations of hexadecyltrimethylammonium bromide (HDTMA) or benzalkonium chloride (BAC) can be readily adsorbed onto negatively charged NZ surfaces. The antibacterial activity of such surfactant-modified zeolites (SMZ) has been reported. The NZ modified with BAC cations efficiently removed clinical isolates of *A. baumannii* in a physiological solution, with an MBC of 250–500 mg/L [34]. When testing different samples of NZ modified with HDTMA cations against the phosphate-accumulating bacterium *Acinetobacter junii* in simulated wastewater, an antibacterial activity ranging from 0 to 100% was obtained [60]. This phenomenon was explained by the surface configuration of cationic surfactant molecules sorbed onto NZ. Particles of SMZ exhibit bactericidal action, but only in cases of bilayer or patchy bilayer coverage of NZ particles. In the case of a monolayer configuration of cationic surfactant, SZM functions as an antibacterial agent (reducing the concentration of viable bacteria compared to the positive control). If cationic surfactant molecules are sorbed onto NZ in partial monolayer coverage, bacteria remain unaffected [63]. Measuring the zeta potential of SMZ particles helps predict antibacterial activity. The zeta potential of particles below the isoelectric point suggests the sorbed surfactant exists as monomers or hemimicelles. In contrast, the zeta potential above the isoelectric point indicates the sorbed surfactant forms a patchy or complete bilayer. Changes in the zeta potential of SMZ particles are accompanied by changes in the occupied adequate cation exchange capacity (ECEC) of NZ (Figure 4). When using SMZ, special attention should be given to the concentration of desorbed surfactant molecules, as they can be toxic to all aquatic organisms [64].

Thymol and carvacrol are monoterpene phenolic derivatives of cymene with distinct structural configurations. Despite their structural disparities, they exhibit unique physicochemical properties, biological activities, and environmental behaviors [65]. The growing preference for natural products over harmful synthetic alternatives has led to the widespread use of essential oils rich in bioactive compounds like thymol and carvacrol. A recent study on the ecotoxicity of these two structural isomers, focusing on critical indicators in aquatic and soil ecosystems as well as microbial communities from rivers and soils, shows that both compounds display acute toxicity towards the four indicators, surpassing the toxicity levels reported for certain antibiotics [66]. Soil microorganisms revealed greater sensitivity than aquatic microorganisms, with carvacrol demonstrating higher toxicity than thymol [66].

One method to harness the beneficial bioactive characteristics and mitigate or even eradicate adverse environmental effects is to immobilize bioactive compounds on sustainable supports. Thymol and carvacrol can be immobilized on the surface of NZ via supercritical solvent impregnation using supercritical carbon dioxide (CO_2_) [67]. The content of both phenols in NZ composites was approximately 20 wt.%. They demonstrated antibacterial efficacy against *E. coli* DSM498 and *S. aureus* ATCC25923 in various aqueous environments (phosphate buffer solution, spring water, and lake water). Their activities varied, and after one hour, the carvacrol-infused composite exhibited superior antibacterial efficacy compared to the thymol-infused composite. Both composites showed a bactericidal effect after 24 h of contact. The leaching of phenols from the composite in water was more significant for thymol than for carvacrol (6.2–8.9%), indicating the impact of steric effects on the desorption process [67].

Furthermore, adsorbents based in NZ saturated with ciprofloxacin and salicylate demonstrated significant antibacterial activity against *E. coli*, indicating a potential application of waste adsorbents in the tertiary stage of water treatment [68,69].

As an abundant natural polysaccharide, chitosan has garnered significant attention in biomaterials due to its biocompatibility, biodegradability, and non-antigenicity. Zeolite/chitosan hybrid composites can be easily adjusted for zeolite contents and shapes, while also possessing high porosity and mechanical strength. The compressive mechanical strength increases with higher zeolite content [70]. The functionalization of zeolite with chitosan results in chitosan-coated zeolites that are suitable for biomedical applications. Caffeic acid can be effectively grafted onto chitosan using laccase, endowing the material with antioxidant characteristics. The immobilization of glucose oxidase transforms the zeolite particles into an in situ hydrogen peroxide generating system, producing the oxidative agent and demonstrating antibacterial efficacy [71].

## 4. Practical Application of Modified NZ in a Real Environment

Unlike the controlled conditions in laboratory-scale experiments, the actual environment is characterized by fluctuations in ecological factors. Therefore, applying functionalized NZ in the natural environment poses challenges. NZs have been employed in the Chernobyl (1986) and Fukushima Daiichi (2011) nuclear disasters to reduce contamination from Cs-137 and Sr-90 radioactive ions [72]. With hundreds of thousands of tons used, these areas represent the largest in situ applications of NZs. The applications of NZ for bacterial control in the environment have not been performed on such a large scale. Most studies evaluated the efficacy of functionalized NZ for bacterial control in actual water samples, whereas fewer focused on soils [73].

In the effluent from a secondary wastewater treatment plant (WWTP), 1 wt% Cu- or Zn-exchanged NZ reduced the numbers of artificially added *E. coli* DSM498 and *S. aureus* DSM799 by six log within 24 h of contact [32]. Similar efficiency was achieved by adding NZ infused with Cu_2_O or ZnO nanoparticles. Furthermore, 2.2 log of the native population of *E. coli* in nonsterile effluent water was completely removed after 1 h of contact by adding 1 wt% of Cu_2_O or ZnO functionalized NZ [41]. A 0.1 wt% of Cu(II)-exchanged NZ removed 107 CFU/mL of *E. coli* DSM498 and environmental isolates of *E. coli* from lake water and spring water after 6 h of contact [32].

**Figure 5:**
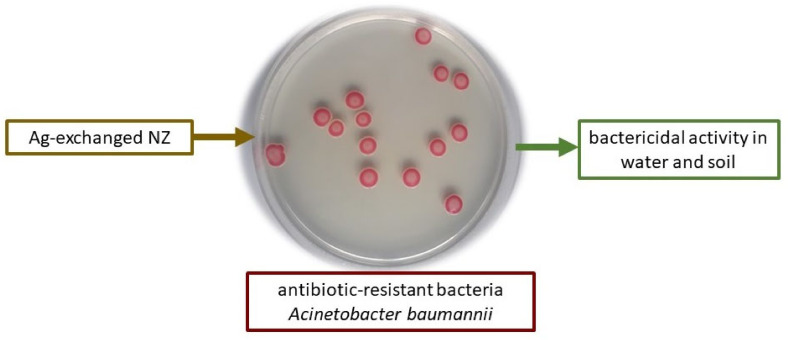
Colonies of a pandrug-resistant environmental isolate of *Acinetobacter baumannii* grown on CHROMagarTM *Acinetobacter*. The photograph was taken in the Faculty of Science, Division of Microbiology laboratory in Zagreb. Viable bacteria contaminating natural water or soil can be controlled by adding Ag-exchanged NZ.

Although there is no significant difference in the antibacterial activity of functionalized NZ against non-sporogenic bacteria, studies involving emerging pathogenic bacteria are essential to validate the concept. A limited number of studies have been conducted on clinically relevant antibiotic-resistant bacteria, such as *A. baumannii* (Figure 5). A pandrug-resistant environmental isolate of *A. baumannii* inoculated into natural spring water was effectively removed by up to 5.6 log in a bead filter system filled with Ag-exchanged NZ. In the same column setup, all clinically relevant carbapenem-resistant bacteria were removed from WWTP effluent [33].

The Cu(II)- and Ag-exchanged NZ were tested for remediation of soils artificially contaminated with 6.7 log CFU/g of an environmental isolate of *A. baumannii* [74]. Adding 1 wt% of Cu(II)-exchanged NZ removed all *A. baumannii* within three days in slightly acidic terra rossa and within 14 days in slightly alkaline red paleosol. Adding only 0.1 wt% of Ag-exchanged NZ was sufficient to eliminate *A. baumannii* within 1 h of contact from both soils. Spore-formers dominated the population of native heterotrophic bacteria in soils and were thus not influenced by the antibacterial activity of metal-exchanged NZ [74]. Although the functionalized NZ can demonstrate excellent bactericidal activity against potentially pathogenic bacteria, we must remember that this does not apply to bacterial spores. Namely, spores of pathogenic bacteria, such as *Clostridium* sp., exhibit significant resistance to toxic heavy metals and bactericidal organic substances [75].

Soil acts as a filter and reservoir for pathogenic bacteria, reducing their contamination of groundwater resources. As a transporting agent, the rate and extent of bacterial movement are influenced by water flow, whether from irrigation or rainwater [76]. Pathogens can leach into groundwater even after manure application [77,78]. It is estimated that around 75% of the antibiotics used in cattle production are excreted in animal waste rather than absorbed by the animals [79]. Consequently, antibiotic resistance can develop among gastrointestinal bacteria, such as *E. coli*, which are also expelled in manure and can pollute the environment [80].

Soil factors, such as soil type, pore size, structure, soluble organic carbon, and colloidal carriers, facilitate the transport of bacteria to water resources. Animal manures contain organic colloids similar in size to bacteria, which increases the risk of bacterial contamination in water resources [81]. Research has shown that nano NZ can reduce heavy metal uptake into plant root systems [82] and mitigate the impact of fecal bacteria in the soil by preventing their transport [83].

Depending on the soil’s composition and quality, zeolite can alter its porosity, the distribution of pore sizes, the connectivity of the pores, and the tortuosity. The infiltration rate, the fraction of water that saturates with hydraulic conductance, the capacity for water retention, and the management of nutrients that seep into the soil are all influenced by zeolite [84]. NZ can enhance soil health and productivity, potentially offering a new solution to problems related to water and nutrient deficiencies in the soil. Recently, researchers have investigated NZ for its ability to suppress the production of biogenic amines. Biogenic amines are found in a wide range of foods, and their accumulation is associated with bacterial proliferation that contributes to food spoilage [85]. Studies show that 1–5 wt.% NZ can reduce biogenic amines and ammonia production by Gram-positive and Gram-negative bacteria, with *E. coli* and *Aeromonas hydrophila* being the largest amine producers. This effect is attributed to the direct influence of NZ on microbial growth [85].

### Health Protection

**Figure 6:**
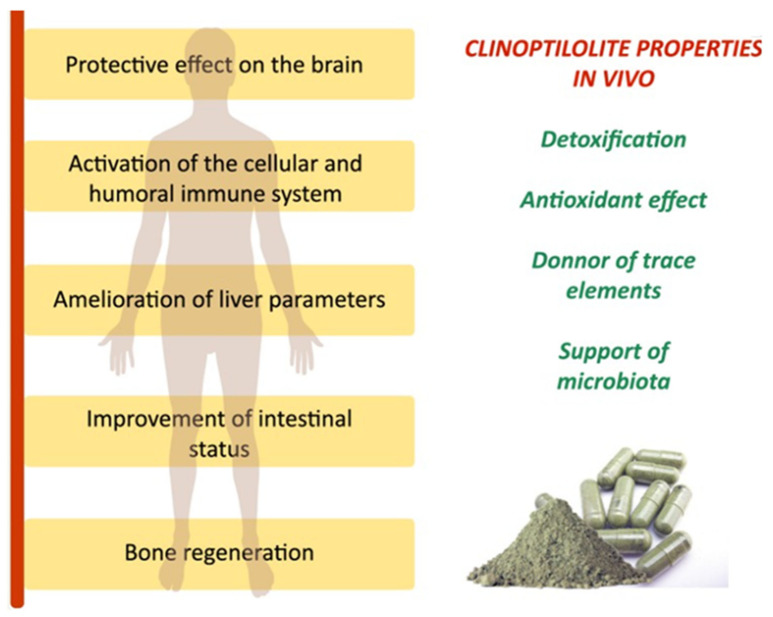
Clinoptilolite materials have been shown in animal and human studies to have clinical benefits when supplemented in diet powder form [87].

NZ has been reported to effectively capture and remove a wide range of compounds, both in vitro and in vivo, including microbial toxins, medicines, biochemicals, and viruses [86]. Accordingly, clinoptilolite-based materials have demonstrated beneficial effects on the health of both animals and humans due to their detoxifying properties, along with their ion-exchange and adsorption capabilities (Figure 6).

These materials have been shown to aid in removing contaminants and promoting intestinal health [87]. However, the specific applications and benefits of these materials in humans require thorough investigation.

It is worth noting that using NZ to prepare chocolate and biscuits for cesium decontamination in children following the Chernobyl disaster is significant due to its resilience in acidic environments and pronounced selectivity for certain cations. Experiments show that the structure of clinoptilolite is not substantially altered by stomach fluids, and the purified clinoptilolite is suitable for formulating systems that utilize its properties as a pharmaceutical excipient [88].

**Figure 7:**
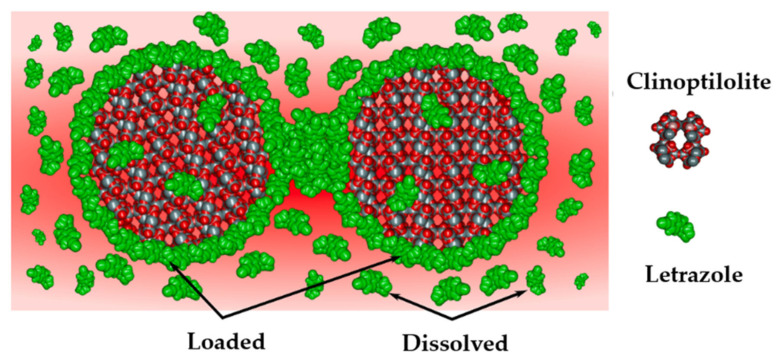
Dissolution of the letrazole molecules from the clinoptilolite surface [89].

Currently, various NZ-containing materials are being studied for medical applications. These materials contain trace elements and are pre-loaded with several cations. Therefore, interactions between drugs and porous carriers play key roles in the drug adsorption and desorption kinetics. Letrozole acts as an aromatase inhibitor and is used for breast cancer treatment in women; however, its solubility and dissolution are quite poor. It has been shown that the dissolution of letrozole is three times faster than that of the bulk drug when delivered through desorption from clinoptilolite (Figure 7) [89].

The surfactant-modified NZ was also tested as a diclofenac support. The composite exhibits a rapid initial diclofenac release but maintains a gradual release that is suitable for prolonged administration after 6 h [90].

It is important to note that purified clinoptilolite has been approved by the Cuban Drug Quality Agency as an anti-diarrheal human drug [91]. *Helicobacter pylori* is a bacterium whose pathogenic strains cause severe gastroduodenal diseases. Ammonium plays a crucial role in the survival of *H. pylori* and enhances the effect of a toxin produced by the bacterium. In vitro tests showed that Na-clinoptilolite decreases bacterial growth by 13 to 87% for Na-clinoptilolite concentrations ranging from 0.5 to 8 mg/mL [92]. The effect should be ascribed to the results of in vivo experiments with rats. It was evidenced that clinoptilolite binds ammonium ions in the upper gastrointestinal tract [93], thereby inhibiting the growth of *H. pylori*.

Chronic wounds are a significant global healthcare issue, affecting 0.18–2% of the general population and up to 5% of individuals over 65. Clinoptilolite-based products demonstrate promising results in wound treatment and are safe and well tolerated in rodents. Reports indicate that topical purified NZ application is safe for treating acute wounds in healthy male volunteers, as no adverse events were noted throughout this phase I clinical trial [94]. Moreover, malignant fungating wounds produce pain, pruritus, and odor due to bacterial byproducts, including cadaverine and putrescine. A micronized pure clinoptilolite-tuff formulation was rapid, stable, and effective in absorbing cadaverine. Clinoptilolite-based products have been shown to be safe and well tolerated [95]. Additionally, the hemostatic efficacy and wound healing characteristics were compared in a lethal rabbit model of complicated groin injury. The use of natural clinoptilolite markedly reduced mortality and expedited the wound healing process. Due to its low cost, ease of manufacture, and excellent biocompatibility, it is recommended as a superior alternative to synthetic zeolite-type hemostatic treatments, such as the hemostatic agent QuikClot, which contains synthetic zeolite 5A [96].

The films made from gelatin, glycerol, and Ag-clinoptilolite exhibited suitable properties for wound dressings, as the findings aligned with several essential desirable attributes for such applications. The gradual release of silver ions demonstrates their antibacterial function, making them appropriate for use in wound dressings. The antimicrobial assay results indicated that films containing Ag-clinoptilolite showed effective antibacterial action, as evidenced by the inhibitory halo surrounding the films tested against *S. aureus* and the indigenous microorganisms on human skin [97].

**Figure 8:**
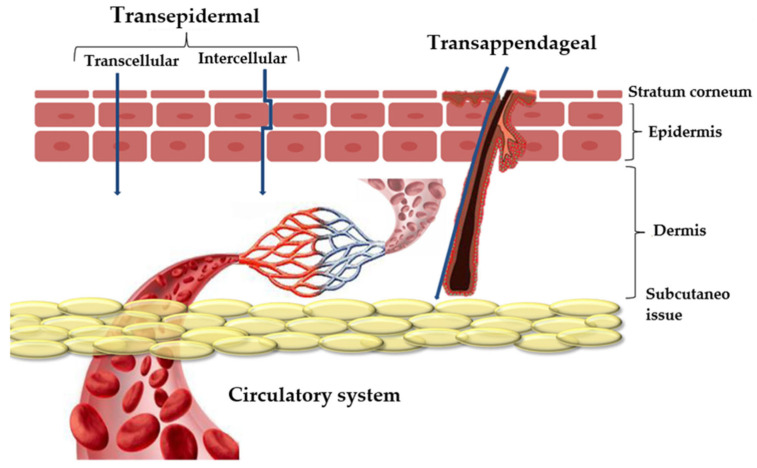
Layers of skin and several pathways of drug penetration [98].

Skin drug delivery can provide local or systemic therapeutic effects. The two main routes of penetration are transappendageal and transepidermal (Figure 8). The transappendageal route is preferred by ions and large polar molecules, while the transepidermal route involves two pathways: intracellular (through corneocytes) and intercellular (through a tortuous path within the continuous lipid domain) [98]. Although the exact mechanisms are still under investigation, clinoptilolite most likely exchanges ions with the wound milieu, releasing beneficial ions such as calcium and sodium, which are important for cellular functions and tissue regeneration. It can also bind toxins and exudates, reducing local inflammation and promoting healing.

Modifying cotton (through mercerization) and polyester (via alkaline hydrolysis) fabrics for summer clothing, with the addition of NZ nanoparticles, yields UV and antibacterial protective textiles. Textiles and clothing containing clinoptilolite nanoparticles can shield the skin, demonstrating effects on the healing process of skin wounds and therapeutic benefits against various skin diseases such as psoriasis and cancer [99].

## 5. Factors That Influence the Antibacterial Activity of Functionalized NZ

Estimating the antibacterial activity of functionalized NZ requires selecting bacterial species that are native or likely to exist in the target environment. Attention should focus on the specific strain used to target pathogenic bacteria. Bacterial strains stored in microbial banks often exhibit genetic and physiological differences from undesirable environmental strains. Experiments involving consortia of various bacterial species are also advised. While a model bacterium for estimating the antibacterial activity of functionalized NZ is currently unavailable, it can be inferred that antibacterial activity is relatively consistent across bacterial species, as long as non-spore-forming bacteria are studied. Most data indicate the antibacterial activity of functionalized NZ against non-sporogenic, Gram-positive, and Gram-negative aerobic bacteria. Simultaneously, there is a lack of research examining antibacterial activity against anaerobic bacteria in environmental contexts [100].

Functionalized NZ is generally prepared by modifying NZ with agents that demonstrate broad antibacterial activity. As a result, the antibacterial activity of functionalized NZ is also nonspecific to particular bacterial species or strains. In environmental samples, the selection of antibacterial activity can occur due to the natural resistance and fitness of specific species compared to others within the ecological population [75].

As with any antibacterial agent, the effectiveness of functionalized NZ depends on various ecological factors, such as pH level, temperature, and the availability of water or nutrients for heterotrophic bacteria. Generally, bacteria are sensitive to acidic (pH below 5) or alkaline (pH above 9) conditions, making it unlikely for human pathogens to multiply in such environments. Pathogen survival is significantly better at low temperatures (below 4–22 °C) than at higher temperatures (35–44 °C) [101]. A layer of water surrounding a non-sporogenic bacterial cell is essential for its survival and serves as the medium for transferring antibacterial molecules from functionalized NZ to bacterial cells. Therefore, in a dry environment, the antibacterial activity of functionalized NZ is not anticipated; however, non-sporogenic bacteria will still deteriorate.

The antibacterial activity of functionalized NZ is highly dependent on the nutrient concentration in the medium. The higher the nutrient concentration, the weaker the antibacterial activity [55]. This fact is well known in the application of commercial disinfectants and antiseptics, where organic matter should be removed before use.

## 6. Conclusions

It is widely accepted that aluminosilicate zeolites possess exceptional ion exchange characteristics, enabling effective utilization as reservoirs for antimicrobial metal ions and carriers of tiny nanoparticles. It has been demonstrated beyond a reasonable doubt that the zeolitic open-framework structure enhances nanoparticle stability and increases the efficiency of their distribution. Despite the negatively charged surface of zeolites, this charge can be altered and optimized, facilitating the functionalization of the surface with various antimicrobial agents. Much research has been conducted on the antibacterial properties of zeolites, leading to the development of numerous concepts for producing functional composites. The antibacterial mechanisms of composites based on zeolites have been proposed, with the primary mechanism being the in situ release of transition metal ions known for their antimicrobial characteristics, primarily silver, copper, and zinc. This is made possible by the microporous frameworks, which allow for the rapid movement of metal ions within the particles and significant discharge into the surrounding environments. Although many tests have been conducted, the release kinetics and the amount of active species liberated remain inadequately regulated. In light of this, it is imperative to conduct a more in-depth investigation into utilizing these readily accessible natural materials, particularly in real-world scenarios and with authentic samples. To compare the outcomes of different studies, it is essential to understand that establishing a standardized technique for evaluating the effectiveness of antibacterial agents is crucial. The opportunities for exploiting natural microporous materials in water treatment facilities and soil remediation will increase.

Natural clinoptilolite and its modified variants demonstrate considerable potential in health preservation, particularly for detoxification and antioxidant capabilities. Adhering to toxins and pathogens enhances a more favorable intestinal environment, which is essential for comprehensive immune activity. The efficacy of clinoptilolite in wound healing and skin care, along with its protective characteristics against UV radiation, makes it highly promising for various applications in global environmental protection.

In conclusion, even though zeolites are generally considered innocuous, it is vital to remember that the long-term effects of nanoparticles on ecosystems and their species remain largely unknown. This should be taken into account. Additionally, thorough risk assessments and environmental monitoring are necessary to ensure that nano-zeolite is safe and sustainable. Another important consideration is that the use of nanomaterials, such as nano-zeolite, may be subject to specific guidelines and limits.

## Data Availability

No new data were created or analyzed in this study.
